# Melatonin reduces IL‐33 and TSLP expression in human nasal epithelial cells by scavenging ROS directly

**DOI:** 10.1002/iid3.788

**Published:** 2023-02-16

**Authors:** Min‐jie Gong, Hai‐bao Zhang, Miao Lou, Yu‐sheng Wang, Rui‐ping Ma, Zhen‐zhen Hu, Guo‐xi Zheng, Ya Zhang

**Affiliations:** ^1^ Department of Otolaryngology Head and Neck Surgery The Second Affiliated Hospital of Xi'an Jiaotong University Xi'an Shaanxi China; ^2^ Oncology Research Lab, Key Laboratory of Environment and Genes Related to Diseases, Ministry of Education Xi'an Jiaotong University Xi'an Shaanxi China

**Keywords:** Chronic rhinosinusitis, IL‐33, Melatonin, Nrf2, ROS, TSLP

## Abstract

**Background:**

Chronic rhinosinusitis (CRS) is a chronic mucosal inflammation of the nasal cavity and sinuses. It is classified into CRS without nasal polyps and CRS with nasal polyps (CRSwNP). CRSwNP has high recurrence, especially CRSwNP with massive eosinophil infiltration which is mediated by type 2 inflammatory response. Melatonin is a hormone secreted by the pineal gland, it has powerful antioxidant and anti‐inflammatory effects in addition to regulating biological rhythms. There are no studies on melatonin for the treatment of CRS, so we aimed to explore whether melatonin could be used for the treatment of CRS.

**Materials and Methods:**

In this study, we used melatonin to treat a cell model of CRS. Subsequently, MTT assay was performed to examine the cell viability of human nasal epithelial cells (HNEpCs), a reactive oxygen species (ROS) kit to detect ROS production, a malondialdehyde (MDA) kit to detect the MDA content in the cell culture supernatant, and an apoptosis kit and Western blot analysis to detect apoptosis. The expressions of Nrf2, HO‐1, IL‐33, TSLP, and IL‐25 were detected by Western blot analysis.

**Results:**

Melatonin improved the viability of HNEpCs, reduced lipopolysaccharide‐induced ROS, reduced the MDA content, and inhibited their apoptosis. More importantly, melatonin reduced the expression of IL‐33 and TSLP, an important phenomenon for the treatment of CRSwNP.

**Conclusion:**

Melatonin protects HNEpCs from damage in inflammation and reduces IL‐33 and TSLP expression of HNEpCs.

## Graphical abstract

Melatonin attenuates inflammatory damage in human nasal epithelial cells (HNEpCs) by directly scavenging ROS. Melatonin reduces IL‐33 and TSLP expression in HNEpCs.

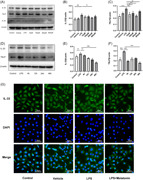

## 1 INTRODUCTION

Chronic rhinosinusitis (CRS) is a chronic and persistent inflammatory disease of the nasal mucosa. It manifests as nasal congestion, runny nose, head and facial pain, and a reduced sense of smell occurring for more than 12 weeks,^1^ with a prevalence of approximately 1.1%–4.3% worldwide.^2^ It is broadly classified into CRS with nasal polyps (CRSwNP) and CRS without nasal polyps.^3^ CRSwNP is highly heterogeneous and involves multiple immune cells. It is divided into type 1 immune responses involving Th1, type 2 immune responses involving Th2, and type 3 immune responses involving Th17 based on the differentiation of Th cells in polyps.^4^ Most Caucasian and half of Asian patients diagnosed with CRSwNP demonstrate type 2‐biased immune responses, which are profound tissue eosinophilia.^5^ CRSwNP with massive eosinophil infiltration (ECRSwNP) is characterized by type 2 inflammation, with poor treatment outcomes and high recurrence tendency.^6^ Studies postulate that thymic stromal lymphopoietin (TSLP), IL‐25, and IL‐33, secreted by nasal mucosal epithelial cells in response to stimulation by external substances, play an important role in initiating type 2 immune responses.^7^ Glucocorticoids and surgery remain the primary CRSwNP treatment strategies. However, a search for an effective pharmacological treatment remains critical because of the complexity of its pathogenesis, ease of recurrence (especially type 2 inflammatory response‐mediated), and the burden of repeated surgeries.

Melatonin (N‐acetyl‐5‐methoxytryptamine) is a hormone secreted by the pineal gland and is widely known for its role in regulating biological rhythms.^8^ In addition, it exhibits powerful effects in scavenging reactive oxygen species (ROS) and anti‐inflammation.^9^ It can interact with reactive free radicals to scavenge them directly or indirectly by regulating antioxidant proteins, especially Nrf2/HO‐1, a key signaling pathway involved in oxidative stress, to scavenge ROS.^10^, ^11^ Current studies suggest that melatonin reduces inflammatory responses or their adverse effects by mainly reducing oxygen free radicals, promoting antioxidant processes, and producing excess nitric oxide.^12^ Some studies postulate that melatonin has significant potential in treating various diseases because of its powerful antioxidant and anti‐inflammatory effects.^13^, ^14^, ^15^, ^16^, ^17^ However, there are no studies on the use of melatonin in the treatment of CRS.

This study was conducted to determine the protective effect of melatonin on human nasal epithelial cells (HNEpCs) and its scavenging ability on lipopolysaccharide (LPS)‐induced ROS. The ability of melatonin to alleviate LPS‐induced apoptosis in HNEpCs was also investigated. The expression of IL‐33, TSLP, and IL‐25 was subsequently tested to evaluate the protective effect of melatonin on epithelial inflammation. This study provides preliminary clues for using melatonin to treat type 2 inflammation‐mediated CRSwNP.

## 2 MATERIALS AND METHODS

### 2.1 Cell culture

The HNEpCs were purchased from Otwo Biotech. They were subsequently cultured in different plates (depending on the purpose) containing Dulbecco's minimum essential medium (Gibco) supplemented with 15% fetal bovine serum (Gibco) and 1% penicillin (5000 μg/mL)‐streptomycin (5000 IU/mL) solution. The culturing was done at 37°C in a 95% humidified air and 5% CO_2_ incubator. Melatonin was dissolved with dimethyl sulfoxide (DMSO) and diluted to 10, 100, 300, and 600 μM, and then we divided HNEpCs into control, Vehicle, LPS‐treated, and melatonin‐treated groups. The control group was not treated with drugs, the Vehicle group was treated with DMSO, the LPS‐treated group was treated with 0.1 μg/mL LPS, and the melatonin‐treated group was treated with different concentrations of melatonin (10, 100, 300, and 600 μM) and 0.1 μg/mL LPS together. All drugs were freshly prepared and replaced with new drugs at 24 h, the treatment time was 48 h. After determining the appropriate melatonin concentration, we treated the cells with melatonin at different times (4, 12, 24, and 48 h).

### 2.2 MTT tests

Cell viability was determined using the 3‐(4,5‐dimethylthiazol‐2‐yl)‐2,5‐diphenyltetrazolium bromide (MTT) assay. HNEpCs (5000) were cultured in 96‐well plates at 37°C and 5% CO_2_ for 24 h, and then treated with DMSO, LPS (0.1 µg/mL), LPS and different concentrations of melatonin for 24 h. After each treatment, 20 μL of MTT solution (5 mg/mL) was added to each well and incubated at 37°C and 5% CO_2_ for an additional 4 h, followed by the addition of 200 μL of DMSO to dissolve the formazan crystals. The absorbance of the samples was then measured at 570 nm using a plate reader (Thermo Fisher Scientific), and data were expressed as mean of optical density ± SD.

### 2.3 Apoptotic and necrotic cells tests

Cells (2 × 10^5^) were seeded in 48‐well plates and cultured at 37°C and 5% CO_2_ for 24 h. They were then treated with DMSO, LPS (0.1 µg/mL), LPS and melatonin (300 µM) for 48 h. The medium was then removed, and the cells were washed thrice with PBS. The apoptotic and necrotic cell tests were conducted following the manufacturer's instructions. Annexin V‐mCherry Binding Buffer (194 μL), 5 μL Annexin V‐mCherry, and 1 μl SYTOX Green were added to each well, followed by 20 min of incubation in the dark and detection using a fluorescent microscope.

### 2.4 Intracellular ROS and malondialdehyde assays

ROS was measured using 2′,7′‐dichlorofluorescein diacetate (DCFH‐DA) to check whether esterases could deacetylate them to form nonfluorescent 2′,7′‐dichlorofluorescein (DCFH) inside the cells. Notably, DCFH reacts with ROS to form a fluorescent product DCF. HNEpCs were seeded at a density of 3 × 10^5^/well in a 48‐plate and then were treated with different agents for 24 h. The culture medium was first removed, and the cells were washed three times with PBS to obtain dissociated HNEpCs for the ROS assay. The cells were subsequently incubated with 10 uM DCFH‐DA for 30 min at 37°C. A fluorescent microscope and a fluorescence plate reader were then used to detect the DCF fluorescence intensity at 488 nm for excitation and 525 nm for emission, respectively. Malondialdehyde (MDA) levels in the supernatant were measured following the instructions of the MDA test kit (Beyotime).

### 2.5 Western blot analysis

The cells were harvested after treatment with each agent for 48 h, washed twice with ice‐cold PBS, and then lysed in whole‐cell lysis buffer at 4°C for 30 min. The lysate was then centrifuged at 12,000 rpm/min at 4°C for 25 min to obtain the supernatant. The protein concentration was subsequently determined using the bicinchoninic acid (BCA) Protein Assay Kit (Beyotime). The protein samples (40 μg) were then subjected to sodium dodecyl sulfate/polyacrylamide gel electrophoresis. The primary antibodies used for Western blot analysis included anti‐β‐actin antibody (1:2000, Santa Cruz Biotechnology), anti‐Nrf2 antibody (1:1000, Proteintech), anti‐HO‐1 antibody (1:1000, Proteintech), anti‐BCL2 antibody (1:1000, Proteintech), anti‐BAX antibody (1:2000, Proteintech), anti‐IL33 antibody (1:1000, Santa Cruz Biotechnology), anti‐TSLP antibody (1:300, Bioss), and anti‐IL‐25 antibody (1:500, Bioss).

### 2.6 Immunocytochemistry

HNEpCs (3000) were cultured in 96‐well plates and treated with each agent for 48 h. The cells were then washed with cold PBS and fixed with 4% paraformaldehyde for 10 min, then permeabilized with 0.5% Triton X‐100 for 30 min. The cells were subsequently blocked with BSA5% for 1 h at room temperature and incubated with anti‐Nrf2 and anti‐IL33 antibodies at 4°C overnight. They were then incubated with daylight 488 (conjugated anti‐rabbit) and daylight 488 (conjugated anti‐mouse) IgG for 1 h at room temperature, stained with DAPI, and observed under a fluorescent microscope.

### 2.7 Statistical analyses

All data were analyzed using the SPSS20.0 statistical software and were expressed as mean ± SD for each group. Comparisons between groups were performed using ANOVA, with a *p* < .05 used as the significance threshold.

## 3 RESULTS

### 3.1 Melatonin alleviates LPS‐induced damage

**Figure 1:**
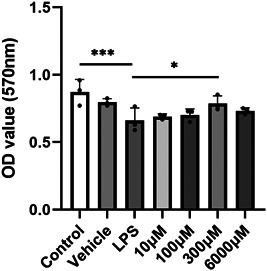
The MTT assay. 300 μM melatonin treatment for 24 h significantly reduced LPS‐induced cell damage, *p < .05, each with *n* = 3 per group, analysis of data using ANOVA. ANOVA, analysis of variance; LPS, lipopolysaccharide.

MTT was used to assess whether melatonin protects HNEpCs from LPS‐induced injury, our results showed that 0.1 μg/mL of LPS significantly reduced the cell viability of HNEpCs compared to the control group, and although different melatonin concentrations were able to reduce LPS‐induced damage, the effect of melatonin at 300 μM was more pronounced (Figure 1).

### 3.2 Melatonin alleviates oxidative stress induced by LPS

**Figure 2:**
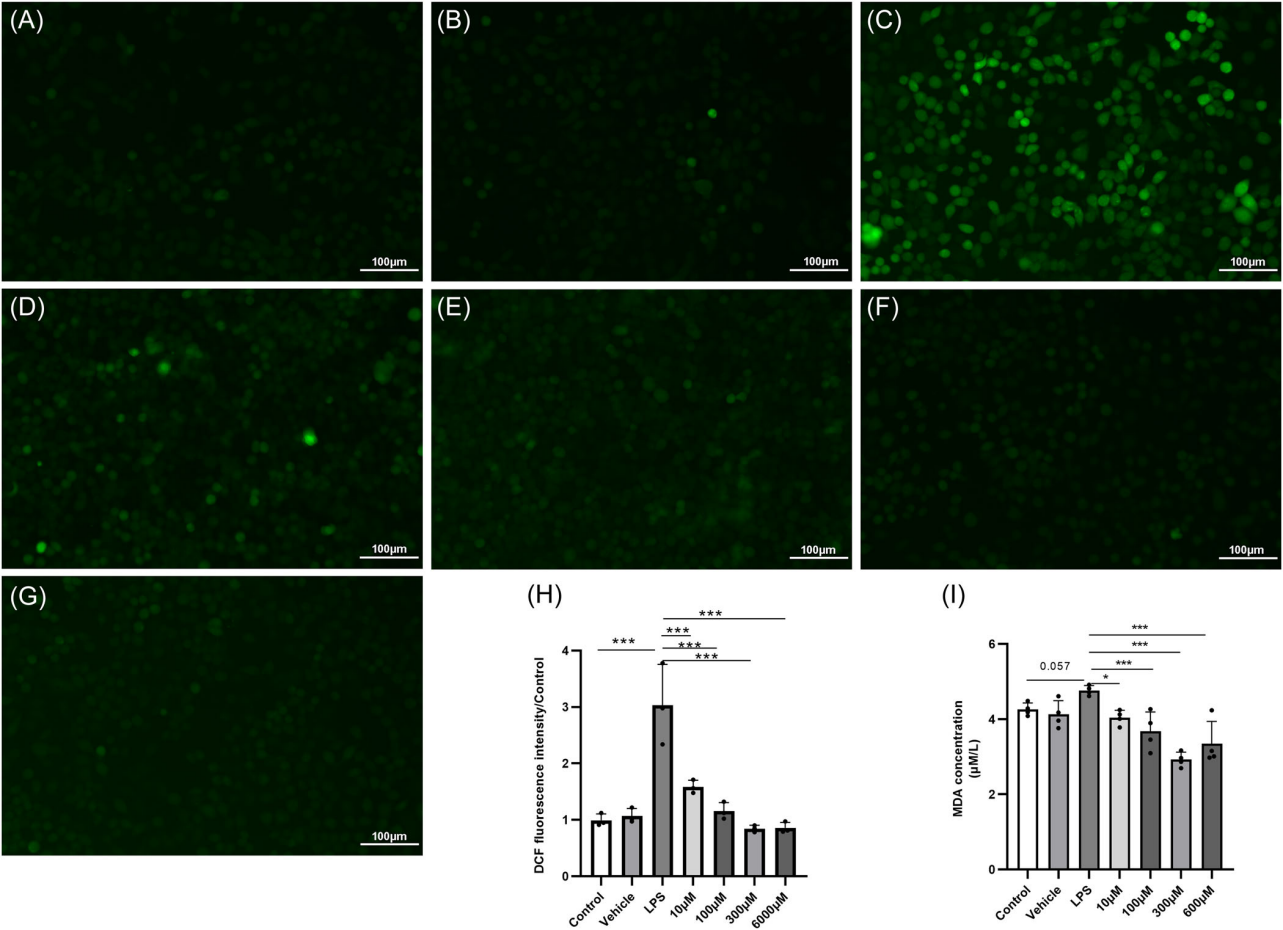
Oxidative stress in cells after 24 h treatment with different drugs. (A–C) Cellular ROS of the control group, vehicle group, and LPS group; magnification, ×20. (D–G) Cellular ROS of 10 μM melatonin concentration, 100 μM melatonin concentration, 300 μM melatonin concentration, and 600 μM melatonin concentration; magnification, ×20. (H) DCF fluorescence intensity of each group, LPS significantly increased the ROS of HNEpCs, ***p < 0.001, different concentrations of melatonin eliminated LPS‐induced ROS, ***p < 0.001, each with *n* = 3 per group, analysis of data using ANOVA. (I) MDA levels in the supernatant. Different concentrations of melatonin significantly reduced MDA levels compared to the LPS group, *p < .05, ***p < .001, each with *n* = 4 per group, analysis of data using ANOVA. ANOVA, analysis of variance; HNEpC, human nasal epithelial cell; LPS, lipopolysaccharide; MDA, malondialdehyde; ROS, reactive oxygen species.

The fluorescence intensity of DCF was used to evaluate the ROS of the cells after 24 h of treatment. LPS caused the cells to produce large amounts of ROS (Figure 2C,H). Notably, different concentrations of melatonin eliminated LPS‐induced ROS (Figure 2D–H). Similarly, after 48 h of treatment, different concentrations of melatonin reduced MDA levels in the supernatant (Figure 2I).

### 3.3 Melatonin reduces allergic cytokines synthesized by nasal epithelial cells

**Figure 3:**
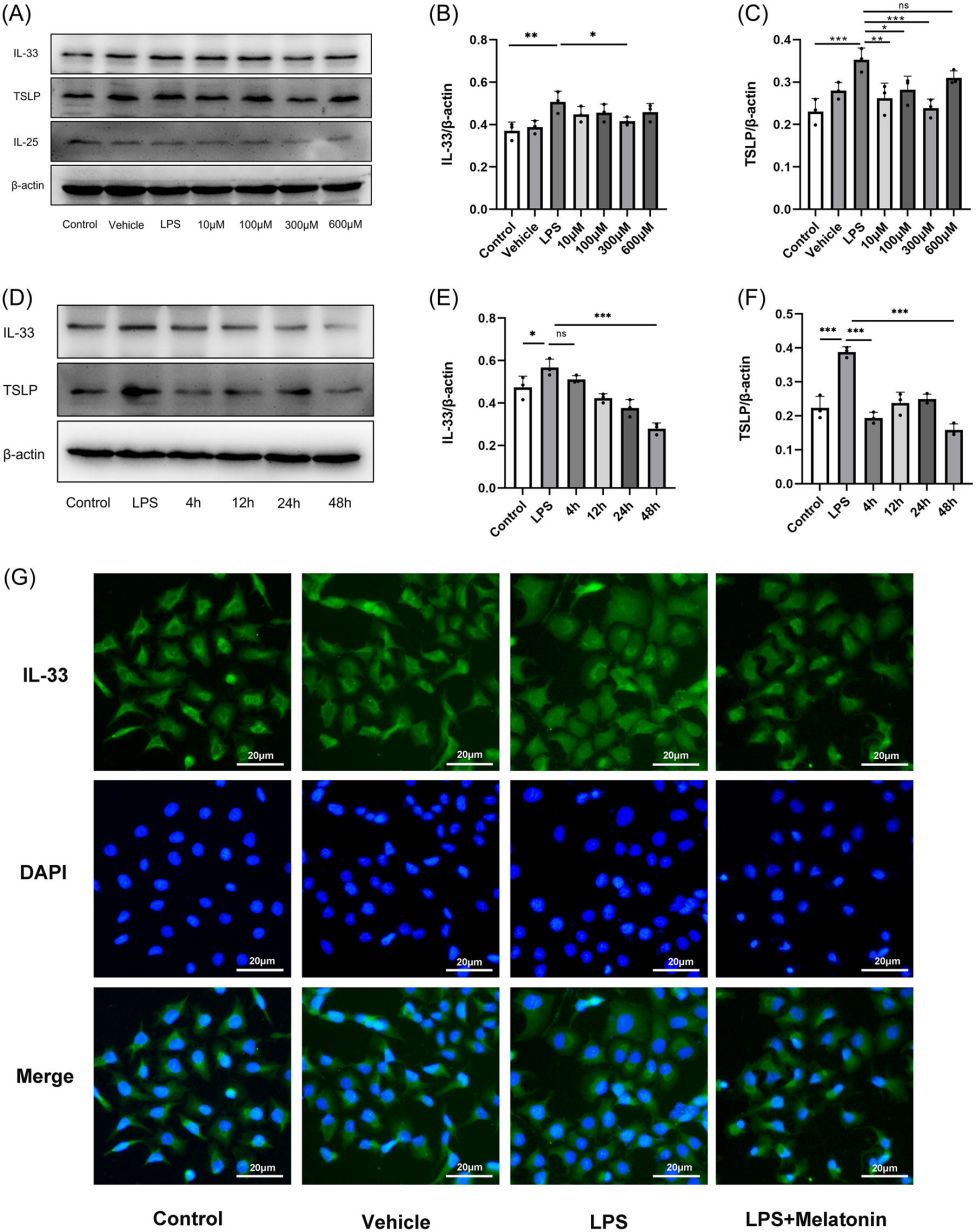
Expression of TSLP, IL‐25, and IL‐33. (A) Expression of TSLP, IL‐25, and IL‐33 in HNEpCs after melatonin treatment at different concentrations. (B, C) Relative expression of TSLP and IL‐33 in HNEpCS after treatment with different concentrations of melatonin, *p < .05, **p < .01, ***p < .001, ns means no statistical significance, each with *n* = 3 per group, analysis of data using ANOVA. (D) Expression of IL‐33 and TSLP in HNEpCs after treatment with 300 μM melatonin for 4, 12, 24, and 48 h. (E, F) Relative expression of IL‐33 and TSLP in HNEpCs after treatment with 300 μM melatonin for 4, 12, 24, and 48 h, *p < .05, ***p < .001, ns means no statistical significance, each with *n* = 3 per group, analysis of data using ANOVA. (G) Immunofluorescence staining of IL‐33 after 300 μM melatonin treatment of HNEpCs, green fluorescence is IL‐33 staining, blue fluorescence is nuclear staining, green fluorescence (IL‐33) is reduced in the cell plasma after melatonin treatment; magnification, ×40. ANOVA, analysis of variance; HNEpC, human nasal epithelial cell.

To investigate whether melatonin regulates IL‐33, TSLP, and IL‐25 expression, we treated HNEpCs with different concentrations of melatonin. The results showed that different concentrations of melatonin reduced IL‐33 expression, but the effect was more pronounced at the concentration of 300 μM (Figure 3A,B). Similarly, different concentrations of melatonin also reduced TSLP expression, but the concentrations of 10 and 300 μM were more effective in reducing TSLP expression (Figure 3A,C). Our results also suggest that melatonin does not regulate IL‐25 expression (Figure 3A). In conclusion, 300 μM is an appropriate concentration for the regulation of IL‐33 and TSLP expression. To further investigate the treatment time of melatonin, we treated HNEpCs with 300 μM melatonin concentration for 4, 12, 24, and 48 h, our results showed that IL‐33 expression decreased with increasing treatment time (Figure 3D,E); however, TSLP expression decreased significantly at 4 and 48 h (Figure 3D,F), we also found that melatonin reduced the transfer of IL‐33 into the cell plasma (Figure 3G). In conclusion, our results showed that melatonin treatment for 48 h was most effective in reducing IL‐33 and TSLP, melatonin treatment for 48 h is the most appropriate treatment time. In summary, our results show that 300 μM melatonin treatment for 48 h is effective in reducing IL‐33 and TSLP.

### 3.4 Melatonin reduces the expression of Nrf2 and HO‐1 induced by LPS

**Figure 4:**
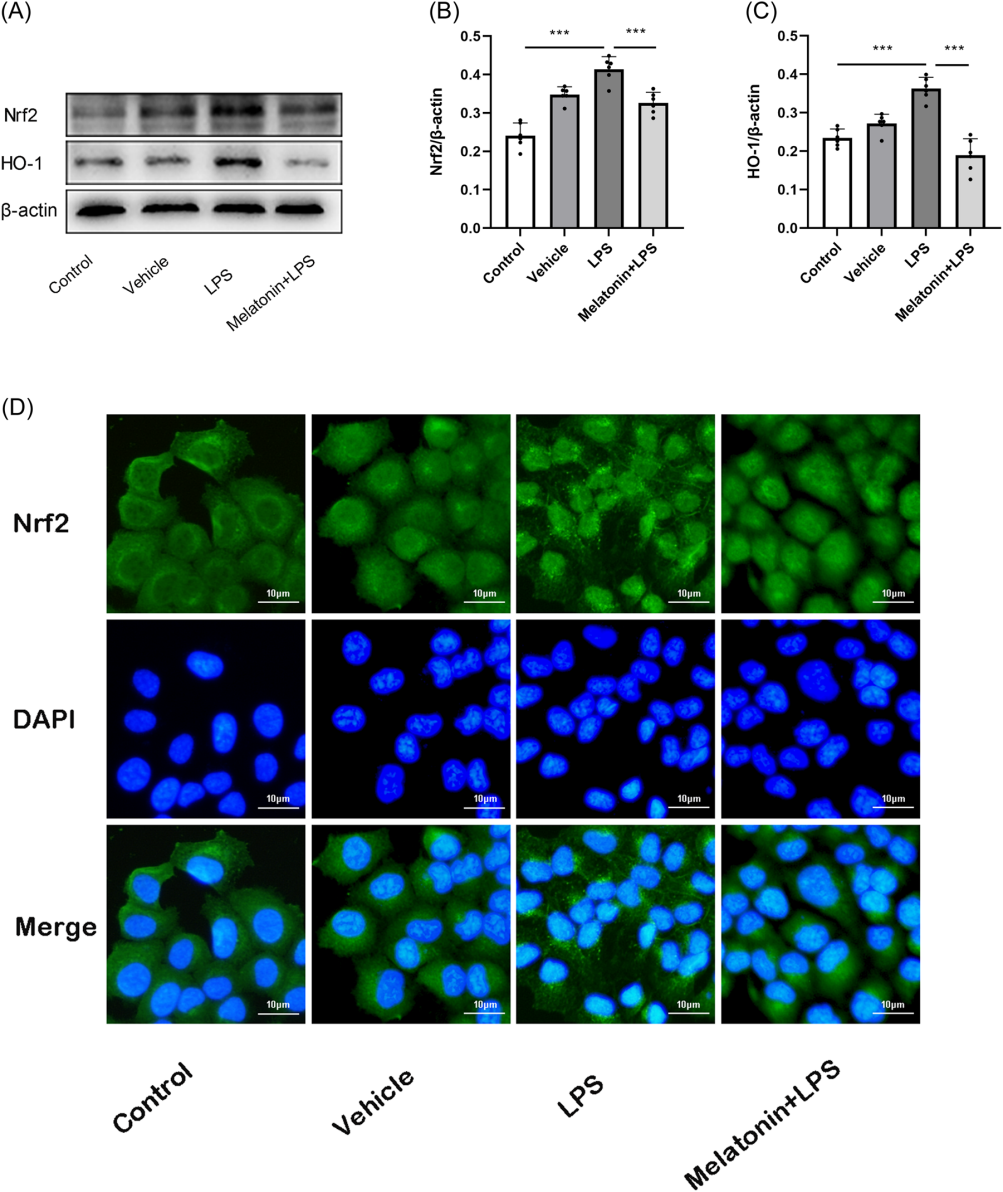
Expression of Nrf2 and HO‐1. (A) Expression of Nrf2 and HO‐1 in HNEpCs after 300 μM melatonin treatment. (B, C) Relative expression of Nrf2 and HO‐1 after 300 μM melatonin treatment, ***p < .001, each with *n* = 6 per group, analysis of data using ANOVA. (D) Immunofluorescence staining of Nrf2, green fluorescence is Nrf2, blue fluorescence is nucleus, melatonin treatment reduces LPS‐induced increase in fluorescence intensity of Nrf2 in the nucleus; magnification, ×40. ANOVA, analysis of variance; HNEpC, human nasal epithelial cell; LPS, lipopolysaccharide.

The core proteins Nrf2 and its downstream protein HO‐1 involved in oxidative stress were extracted after 48 h of 300 μM melatonin treatment, and their expression changes were determined through Western blot. Notably, the expression of Nrf2 and HO‐1 increased after LPS treatment but decreased after 48 h of melatonin treatment (Figure 4A–C). Immunofluorescence results showed that LPS promoted nuclear translocation of Nrf2; however, Nrf2 nuclear translocation was reduced after melatonin treatment (Figure 4D), suggesting that melatonin was able to reverse the activation of Nrf2 by LPS.

### 3.5 Melatonin inhibits LPS‐induced cell apoptosis

**Figure 5:**
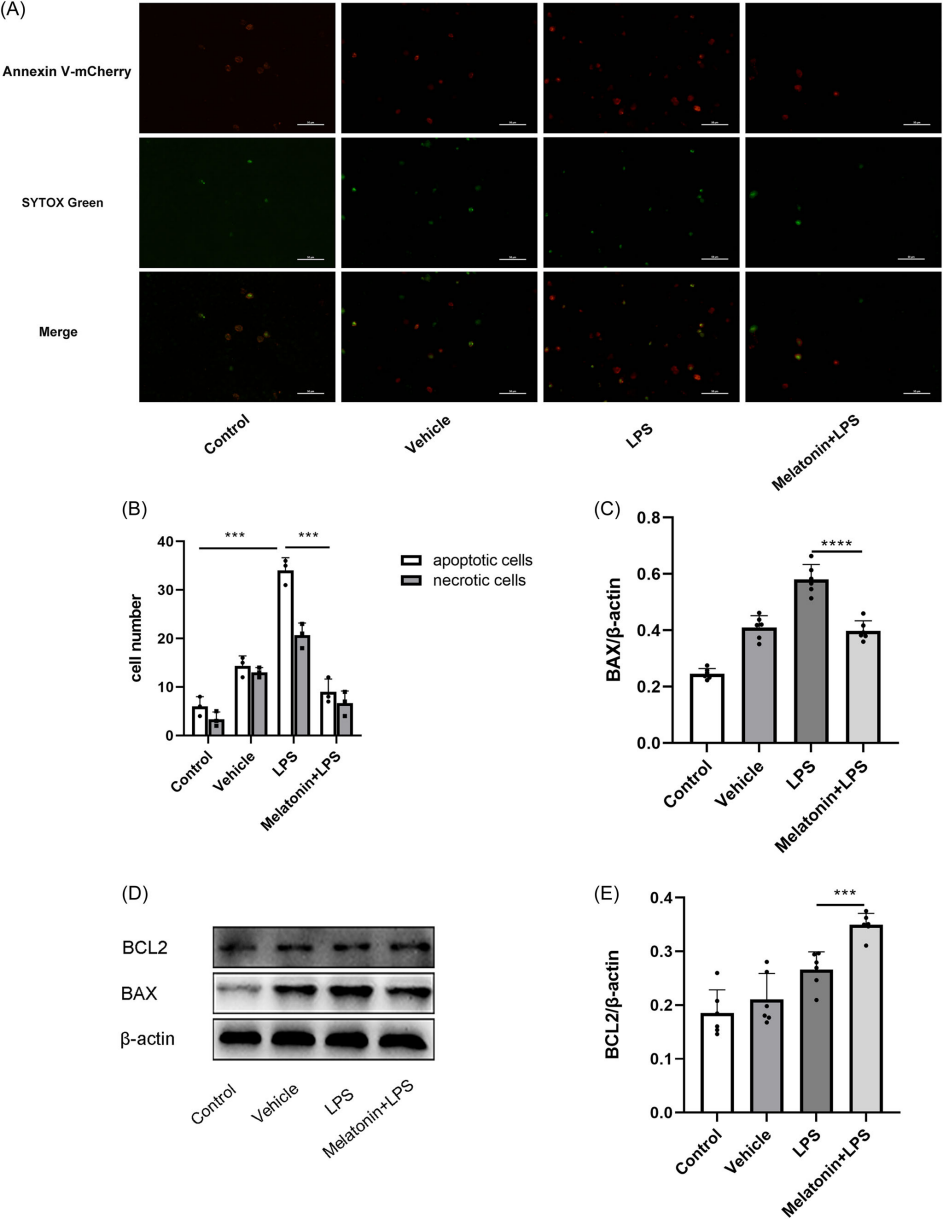
Melatonin treatment attenuates LPS‐induced apoptosis and necrosis. (A) Apoptosis and necrosis staining of HNEpCs, red fluorescence is apoptotic cells, green fluorescence is necrotic cells; magnification, ×20. (B) The number of apoptotic and necrotic cells, 300 μM melatonin treatment for 48 h reduces LPS‐induced apoptosis and necrosis in HNEpCs, ***p < .001, each with *n* = 3 per group, analysis of data using ANOVA. (C) Relative expression of BAX, 300 μM melatonin treatment reduced the expression of the proapoptotic protein BAX compared with the LPS group, ****p < .0001, each with *n* = 6 per group, analysis of data using ANOVA. (D) BAX and BCL2 protein expression after 300 μM melatonin treatment for 48 h. (E) The relative expression of BCL2 was increased by 300 μM melatonin treatment compared with the LPS group for the antiapoptotic protein BCL2, ***p < .001, each with *n* = 6 per group, analysis of data using ANOVA. ANOVA, analysis of variance; HNEpC, human nasal epithelial cell; LPS, lipopolysaccharide.

Apoptosis and necrosis assays were performed to investigate whether melatonin could inhibit LPS‐induced apoptosis. The results indicated that the number of apoptotic and necrotic cells increased after LPS treatment compared with the control group; however, 300 μM melatonin treatment reduced the number of necrotic and apoptotic cells compared to the LPS‐treated group (Figure 5A,B). Moreover, the expression of proapoptotic protein BAX decreased, while that of the antiapoptotic protein BCL2 increased after melatonin treatment (Figure 5C–E).

## 4 DISCUSSION

In this study, 300 μM of melatonin improved cell viability after LPS stimulation of HNEpCs and reduced LPS‐induced intracellular ROS and MDA content generation. Melatonin decreased activation of the Nrf2/HO‐1 signaling pathway, as confirmed by immunofluorescence and immunoprotein blotting. It also inhibited the LPS‐induced apoptosis of HNEpCs. Moreover, melatonin inhibited the synthesis of TSLP and IL‐33, two cytokines that induce the initiation of type 2 inflammatory response in HNEpC. Collectively, the findings of this study suggest that melatonin protects HNEpCs by reducing oxidative stress and the expression of IL‐33 and TSLP.

ROS is a group of molecules formed as a result of incomplete reduction of oxygen. They include superoxide anions (˙O^2−^), hydrogen peroxide (H_2_O_2_), hydroxyl radicals (˙OH), singlet oxygen (1O_2_), peroxy radicals (LOO˙), hydrogen peroxide lipids (LOOH), and peroxynitrite (ONOO^−^) among others.^18^ Low doses of ROS have positive biological effects in maintaining metabolism, signal transduction, cell proliferation, apoptosis, and the aging process.^19^ In contrast, excess ROS can have devastating effects on cells, damaging proteins, lipids, and nucleic acids, causing apoptosis and necrosis in more severe cases.^20^ Normally, cells can scavenge ROS, such as Nrf2, which is the most important. Under normal conditions, Nrf2 binds to Keap1 in the cell plasma to form a biologically inactive molecule degraded by acetylation.^21^, ^22^ However, Nrf2 separates from Keap1 and enters the nucleus to bind at the ARE site when the cell contains a large amount of ROS, thereby promoting the expression of Nrf2 downstream antioxidant proteins HO‐1, superoxide dismutase, and catalase, among others.^23^ In this study, cellular ROS and MDA content increased after LPS stimulation. Nrf2 and HO‐1 expression also increased, indicating that 0.1 µg/mL of LPS stimulation caused oxidative stress in HNEpCs. These findings were consistent with previous studies.^24^, ^25^ The cells were treated with a higher concentration of melatonin, 300 µM, because of its short half‐life and its effectiveness in exerting a direct scavenging effect on ROS when it is available at high local concentrations.^26^ Many studies postulate that melatonin protection against LPS‐induced injury is achieved by activating Nrf2.^27^, ^28^, ^29^ In our study, 300 μM of melatonin did not significantly activate the Nrf2/HO‐1 signaling pathway, a result that is inconsistent with previous studies. Nrf2 is activated and accumulates in cells only when ROS are produced in large amounts. In our case, Nrf2 was not significantly activated yet ROS in the cells was significantly reduced, suggesting that 300 μM melatonin directly scavenged LPS‐induced ROS generation.

Apoptosis is a programmed cell death regulated by a complex set of signaling pathways.^30^ It has an important role in maintaining the homeostasis of an organism's internal environment and embryonic development.^31^ Current studies suggest two main apoptosis pathways: the Death receptor‐mediated apoptosis and Mitochondria‐mediated pathways.^32^ mitochondria‐mediated pathways are the most important pathways for initiating apoptosis in cells. Notably, cytochrome C plays an important role in these pathways.^33^ Under normal conditions, cytochrome C is located in the mitochondria bound to cardiolipin and has an important role in the intracellular electron transport chain reaction.^34^ It is separated from cardiolipin and released into the cytoplasm when cardiolipin is oxidized in the presence of ROS, thereby initiating the apoptosis program.^35^ In this study, 300 μM melatonin directly scavenged LPS‐induced ROS, suggesting that it inhibits apoptosis in HNEpCs by directly scavenging ROS and reducing cytochrome C release, thereby inhibiting cytochrome C‐mediated apoptosis. Apoptosis induced by the mitochondrial pathway involving cytochrome C is controlled by the BCL‐2 family of proteins. These proteins are divided into antiapoptotic (Bcl‐2, Bcl‐X) and proapoptotic (Bax, Bak, Bok, and Bid) classes.^36^ In this study, melatonin increased the antiapoptotic protein BCL2 and decreased the proapoptotic protein BAX, suggesting that it also inhibits ROS‐induced apoptosis in HNEpCs by regulating the BCL2 family proteins. Collectively, melatonin inhibited apoptosis of HNEpCs through two pathways: direct scavenging of ROS and regulation of the BCL2 family proteins.

Melatonin's role in alleviating inflammation is well established. Our study shows that melatonin can reduce the LPS‐induced inflammatory response of nasal epithelial cells and the expression of IL‐33 and TSLP. Currently, the mechanism by which melatonin reduce inflammation is not clear. A previous study reported that melatonin mitigated inflammatory responses by blocking NF‐κB/GSDMD signaling in adipose tissue.^37^ In another study, the mechanism was more complex, involving the PI3K/AKT, ERK, NF‐κB signaling pathway, and mir‐3150A‐3p overexpression.^38^ Notably, numerous studies suggest that melatonin inhibits inflammation by reducing oxidative stress and inhibiting apoptosis and necrosis.^39^, ^40^, ^41^, ^42^ In this study, 300 μM melatonin did not significantly activate Nrf2 but significantly reduced intracellular ROS and MDA levels and inhibited apoptosis, suggesting that melatonin stabilized the LPS‐stimulated epithelial cell state mainly through direct ROS removal, thus inhibiting cell apoptosis and necrosis. IL‐25, IL‐33, and TSLP have recently discovered cytokines that play an important role in ECRSwNP mediated by type 2 inflammatory response.^43^ IL‐33 is localized in the nucleus under basal conditions and is seen as an “alarm” cytokine. It is upregulated and rapidly released outside the cell in response to inflammation or tissue stress.^44^ The findings of this study suggest that melatonin removes the “alarm” effect, thereby reducing the expression of IL‐33. Consistent with previous studies,^45^, ^46^ these results indicate that melatonin inhibits TSLP expression by blocking oxidative stress. In this study, 0.1 μg/mL LPS reduced IL‐25 expression in HNEpCs. However, this phenomenon was reversed by melatonin. In contrast, previous studies report that LPS increases IL‐25 expression in HNEpCs.^47^ Though IL‐33, TSLP, and IL‐25 are epithelial‐derived cytokines that play an important role in allergic airway diseases, the expression of these three cytokines is inconsistent and cross‐talk with each other in chronic airway diseases.^48^ The contrasting expression of IL‐25 from the previous studies may be caused by cross‐talk between the three cytokines. However, further studies are needed to decipher the crosstalk relationship between them.

Despite the insightful findings, this study was limited by several factors. It was an in vitro study. The mechanisms by which melatonin reduces the expression of IL‐33 and TSLP should be further explored despite the strong belief that it is achieved through the reduction of oxidative stress. In the same line, this study did not identify the cross‐talk between IL‐33, TSLP, and IL‐25 after melatonin treatment. Nonetheless, this study provides a new idea for the treatment of CRSwNP mediated by type 2 inflammatory response. It provides a basis for using melatonin in CRS treatment.

## 5 CONCLUSIONS

We report for the first time that Melatonin (300 μM) attenuates LPS‐induced inflammatory response and reduces the expression levels of IL‐33 and TSLP in HNEpCs primarily through direct scavenging of ROS. However, further studies are needed to clarify the limitations of our experiments. This study provides a basis for the use of melatonin for the treatment of type 2 inflammatory response mediated by CRSwNP.

## AUTHOR CONTRIBUTIONS

**Min‐jie Gong**: Conceptualization; investigation; methodology; visualization; writing—original draft. **Hai‐bao Zhang**: Data curation; formal analysis; resources. **Miao Lou**: Resources; validation. **Yu‐sheng Wang**: Visualization; writing—review and editing. **Rui‐ping Ma**: Resources. **Zhen‐zhen Hu**: Data curation. **Guo‐xi Zheng**: Funding acquisition; Supervision; validation. **Ya Zhang**: Funding acquisition; supervision; validation; writing—review and editing.

## CONFLICT OF INTEREST STATEMENT

The authors declare no conflict of interest.

## Data Availability

Some or all data generated or used during the study are available from the corresponding author by request.
